# Anatomical, radiographical and computed tomographic study of the limbs skeleton of the Euphrates soft shell turtle (*Rafetus euphraticus*)

**Published:** 2016-06-15

**Authors:** Behnaz Asadi Ahranjani, Bahador Shojaei, Zahra Tootian, Madjid Masoudifard, Amir Rostami

**Affiliations:** 1 *Department of Basic Science, Faculty of Veterinary Medicine, University of Tehran, Tehran, Iran; *; 2 *Department of Basic Science, Faculty of Veterinary Medicine, Shahid Bahonar University of Kerman, Kerman, Iran; *; 3 *Department of Surgery and Radiology, Faculty of Veterinary Medicine, University of Tehran, Tehran, Iran; *; 4 *Department of Internal Medicine, Faculty of Veterinary Medicine, University of Tehran, Tehran, Iran.*

**Keywords:** Anatomy, Euphrates turtle, Limb skeleton, Radiography

## Abstract

Euphrates turtle is the only soft shell turtle of Iran, and unfortunately is in danger of extinction due to multiple reasons. Imaging techniques, in addition to their importance in diagnosis of injuries to animals, have been used as non-invasive methods to provide normal anatomic views. A few studies have been conducted to understand body structure of the Euphrates turtle. Since there is only general information about the anatomy of turtle limbs, the normal skeleton of the Euphrates limbs was studied. For this purpose four adult Euphrates turtles were used. Digital radiographic examination was performed by computed radiographic (CR) in dorsoventral (DV) and lateral (L) positions. Spiral CT-scanning was done and 3D images of the bones were reconstructed for anatomical evaluation. For skeletal preparation, the skeleton was cleaned by a combination of boiling and mealworm methods and limbs’ bones were examined anatomically. In the present study, simultaneous anatomic, radiographic and CT studies of bones in individual turtles made us possible to describe bones anatomically and provided comparable and complementary conditions to represent the abilities of the radiography and CT for better understanding of the anatomy. Arrangement and the number of carpal and tarsal bones are used in turtles’ classification. Among the studied species, Euphrates turtle carpal and tarsal bones show the most similarities to the *Apolone spinifera*.

## Introduction

Euphrates turtle (*Rafetus euphraticus*) is a species of soft shell turtles that belongs to the class of reptiliane, Testudines order, Trionychidae family, *Rafetus* genus and *R. euphraticus* species.^1^ The species is the only soft shell turtle of Iran, and unfortunately is in danger of extinction.^1^ More and deeper knowledge of such species is essential for its preservation and also choosing appropriate strategies to prevent damages to it. For this purpose, anatomical studies of the body of these animals are proposed as the first step. 

Imaging techniques, in addition to their importance in diagnosis of injuries to animals, have been used as non-invasive methods to provide normal anatomic views, especially in rare animals.^2^^-^^4^ A few studies have been conducted in order to understand body structure of the Euphrates turtle and its differences with other species.^1^^,^^2^^,^^5^ Osteological studies have been conducted on turtle species show anatomic differences between them.^6^ Among these studies, only Taşkavak addressed Euphrates turtle skull morphology and nothing else has been published about its body skeleton.^7^ Since there is only general information about the anatomy of turtle limbs, we decided to study the normal skeleton of the Euphrates limbs. This study as the first study in which normal radiographic and three-dimensional computed tomographic (3D-CT) images of a turtle are compared with the same turtle's bones, also aims to provide researchers with the normal radiographic view of the animal limbs' bones and their 3D position in the body.

## Materials and Methods

**Animals.** In this study, four adult Euphrates turtles were studied with an average weight of 3.5 kg and average carapace (the dorsal part of the turtles’ shell) length of 27.0 cm. The samples were selected from alcohol fixed turtles belonging to the Pars Herpetologists Institute (Tehran, Iran) and transferred to the Department of Surgery and Radiology, Faculty of Veterinary Medicine, University of Tehran, Tehran, Iran.

**Radiologic study.** Digital radiographic examination was performed by computed radiographic (CR) device (Model directView CR 850; Kodak, Rochester, USA) in dorsoventral (DV) and lateral (L) positions. Computed topographic (CT) scanning was achieved using a two slice CT-scan machine (Siemens Somatom Spirit; Berlin, Germany) and 3D images of the bones were reconstructed for anatomical evaluation.

**Anatomical study.** After conducting radiological studies, the samples were delivered to the Department of Anatomy for skeletal preparation. For this purpose, the plastron (the ventral part of the turtles’ shell) and ventral skin of the body were detached. Then, viscera were removed and muscles were separated from the bones as much as possible. The connective tissues and remaining of the muscles were separated from the bones by boiling method. Limbs’ skeleton then situated in a container for one month and their inaccessible parts were cleaned by mealworms from debris. Finally, limbs bones were cleaned and examined anatomically. 

## Results

**Fig. 1:**
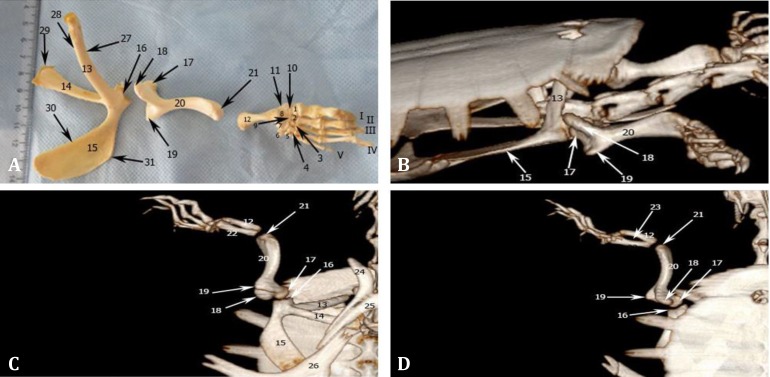
**A)** Dorsal view of bones of the right thoracic limb; **B)** Lateral view of the 3D-CT of right thoracic limb, **C)** Ventral view of the 3D-CT of left thoracic limb; and **D)** Dorsal view of the 3D-CT of left thoracic limb. **1.** 1^st^ Distal carpal (DC), **2.** 2^nd^ DC, **3.** 3^rd^ DC, **4.** 4^th^ DC, **5.** 5^th^ DC, **6.** Pisiform, **7.** Ulnare, **8.** Radiale, **9.** Intermediate, **10.** Centrale, **11.** Distal extremity of radius, **12.** Ulna, **13.** Scapula, **14.** Acromion process, **15.** Coracoid, **16.** Glenoid, **17.** Humeral head, **18.** Dorsal tubercle, **19.** Ventral tubercle, **20.** Humerus, **21.** Distal end of humerus, **22.** Radius, **23.** Interosseous space, **24.** First segment of plastron (epiplastron), **25.** Second segment of plastron (entoplastron), **26.** Third segment of plastron (hyoplastron), **27.** Cranial border of scapula, **28.** Caudal border of scapula, **29.** Medial border of acromion, **30.** Medial border of coracoids, **31.** Lateral border of coracoid. Roman numerals indicate respective digits

**Thoracic girdle. **Thoracic girdle was composed of two bones, the scapula, with its acromion process and the coracoid. The separating border of the two bones was observed only at the glenoid cavity. Glenoid cavity faced cranially and was divided into two unequal parts by a line. The smaller part was observed at the lateral side which was made by the coracoid. The cavity was bean-shaped and its concave part faced dorsally. In terms of location, coracoid and acromion processes were positioned ventrally and scapula was observed dorsally and cranially (Fig. 1A).

Coracoid was consisted of a large wide and a smaller narrow part. The wide part was extended caudally and attached to dorsal surface of the third segment of plastron by soft tissue. The bone had a concave dorsal and a flat ventral surface (Fig. 1A).

Scapula was seen as a V-shaped bone. Its articular surface made the main part of glenoid cavity. Its acromion process was located ventrally and extended medially from each scapula to attach to the first and second segments of the plastron (epiplastrone and entoplastrone), by soft tissue. This process in its most length was seen as a wide cylinder but at the distal part was flattened dorsoventrally (Fig. 1A).

Scapula was extended from the glenoid cavity dorsomedially. It was located in front of the nuchal segment of the carapace where it had a ligamentous attachment. It was flattened dorsoventrally to acquire cranial and caudal borders. Cranial and caudal borders were partly concave and convex, respectively (Fig. 1A).

**Fig. 2:**
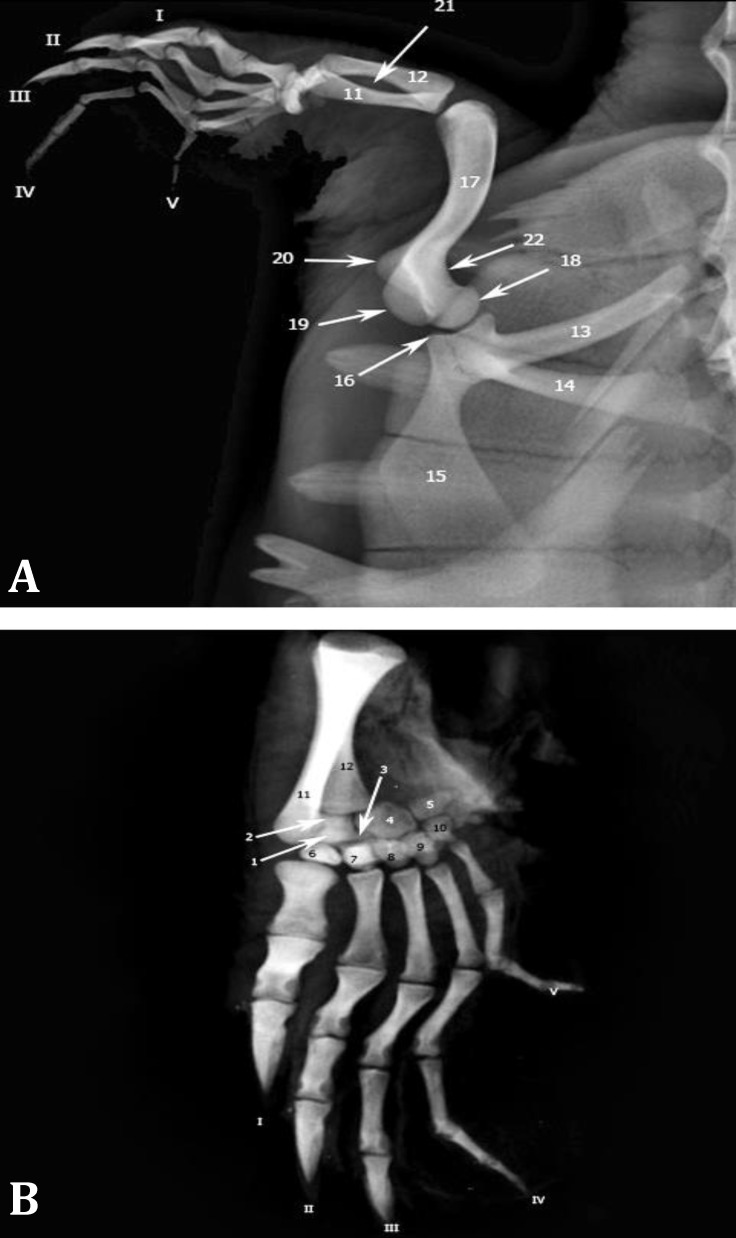
**A)** Dorsoventral radiographic view of left thoracic limb and **B)** Dorsopalmar radiographic view of left autopodium. **1.** Central, **2.** Radiale, **3.** Intermediate, **4.** Ulnare, **5.** Pisiform, **6.** 1^st^ Distal carpal (DC), **7.** 2^nd^ DC, **8.** 3^rd^ DC, **9.** 4^th^ DC, **10.** 5^th^ DC, **11.** Radius, **12.** Ulna, **13.** Scapula, **14.** Acromion process, **15.** Coracoid, **16.** Glenoid, **17.** Humerus, **18.** Humeral head, **19.** Dorsal tubercle, **20**. Ventral tubercle, **21.** Interosseous space, **22.** Neck. Roman numerals indicate respective digits

In DV radiographic view, despite the fact that they had been covered by carapace, the thoracic girdle components were identifiable and extended between second and sixth ribs. Glenoid cavity faced cranially. Scapula and its acromion process according to their connection and anatomic explanation were well-distinguished. Coracoid was not misunderstood with other bones due to its shape and caudal position. The bone at its distal extremity had a superimposition with the third segment of plastron (hyoplastron), (Fig. 2A).

In dorsal view of the 3D-CT images, thoracic girdle components were not observable due to the presence of carapace (Fig. 1D). In lateral view, parts of scapula and coracoid were seen (Fig. 1B). In ventral view, the parts of thoracic girdle which were not covered by plastron were observable. The acromion process was situated more caudally than rod-shaped scapula and extended from the glenoid cavity to the body median line. Some parts of it were covered by first and second segments of plastron (epiplastrone and entoplastrone). Coracoid was extended craniocaudally and located cranial to the third segment of plastron (hyoplastron) where a ligamentous connection attached two bones to each other (Fig. 1C).

**Stylopodium.** In this region S-shaped humerus was extended cranio-caudally parallel to the longitudinal axis of the body. Near the proximal extremity, transverse section of the shaft was almost triangular which gradually was flattened distally, somehow at the distal extremity two dorsal and ventral borders were completely shaped. Articular head was wider and more rose comparing to glenoid cavity. Non-articular dorsal and ventral tubercles were observed on dorsal and ventral sides of the head, respectively. There were two crests along with these tubercles on the humeral shaft. Distal extremity had a wide articular surface. This surface was about twice of the articular surface existing on the radius and ulna proximal extremity (Fig. 1A). 

In DV radiographic view, humerus was observed with its mild S-shaped curvature in front of the glenoid cavity. Articular head was observed clearly with a short distance from the glenoid cavity. Tubercles were recognized in this view. Bone neck was seen medial to the head. Distal extremity was articulated to the zeugopodial bones (Fig. 2A). 

In all three views of the 3D-CT images, the humerus was clearly observed. The bone was located parallel to the body longitudinal axis and its proximal extremity made articulation with the glenoid cavity. Also, the ventral and dorsal tubercles were observed. The shaft was convex medially and concave laterally (Figs. 1B, 1C, and 1D).

**Zeugopodium. **In this region, there were ulna and radius. Two bones were tightly fused proximally and distally to each other and there was a great interosseous space between them. Ulna was wider and shorter than radius. Bones' shafts were narrow in the middle and wide at the ends. Ulna was located dorsal to the radius. Respective greater and lesser proximal extremities of the ulna and the radius made an articular surface for the humerus. This articular surface was smaller than counterpart surface of the humerus. Both bones' proximal extremities were almost located on the same level, but the radius was longer and extended more distally than the ulna. Radius was articulated with the radiale, centrale, and distal carpal I bones and ulna was articulated with the ulnare and radiale of proximal carpal row (Fig. 1A).

In DV radiographic view, both bones were observed. Longer radius was seen caudal to the ulna. Distal extremity of the radius was superimposed with radiale, centrale and intermediate carpal bones. Ulnar proximal extremity was larger than distal one. Due to both bones' curvature, oval interosseous space was seen clearly (Fig. 2A).

**Autopodium.** In this region carpal, metacarpal bones and phalanges were seen. Carpal bones were located in two proximal and distal rows. In each row, five bones were observed. Bones of proximal row had different sizes; the largest bone was ulnare located under ulna; the small pisiforme was connected perpendicularly to the ulnare; the intermediate was located medial to the ulnare and finally radiale was situated under the radius. Small centrale was the most distal bone of the proximal row. Five bones of distal row were located mediolaterally. Each of them was articulated distally with its respective metacarpus. Distal carpal I beside the proximal row, was articulated to the radius distal extremity (Fig. 1A). 

Five long metacarpus bones were consisted of a body and two extremities. Extremities were wider than the shaft. Metacarpal width decreased from I to V and metacarpus III was the longest one. Metacarpal extremities were articulated to their corresponding carpal bone proximally and to the first phalanx distally (Fig. 1A). 

The distal phalanx of digits I, II and III were cone-shaped, ending in a pointed tip. These three digits were out of the flipper. Remaining phalanges showed trochlear articular surfaces on their distal extremities. First to fifth digits had 2, 3, 3, 5 and 4 phalanges, respectively.

In trunk DV radiographic view, autopodial bones were superimposed and not recognizable from each other. To observe these components, positioning was changed and dorsopalmar radiography was done. Resulted radiographs showed all autopodial bones clearly. Regarding to its outermost position, pisiform was seen isolated. Fairly large ulnare was also clearly observed next to pisiform, but the other elements of the proximal row were partially superimposed (Fig. 2B). Five distal carpal bones were distinguished proximal to their correspond matacarpuses. The lines indicating metacapal margins were close together in the middle of the shaft and were apart from each other in the extermities. Metacarpal III was the longest one. Conical processes of the distal phalanges of the 1^st^, 2^nd^ and 3^rd^ digits were observed (Fig. 2B). In the standard views of the 3D-CT images, autopodial components were not identified, therefore, we used software aided rotation to observe them. Proximal carpal row was not clearly distinguished, but all five distal carpal bones were recognized proximal to their correspond metacarpuses. Differential width and length of the bones were well observed.

**Fig. 3:**
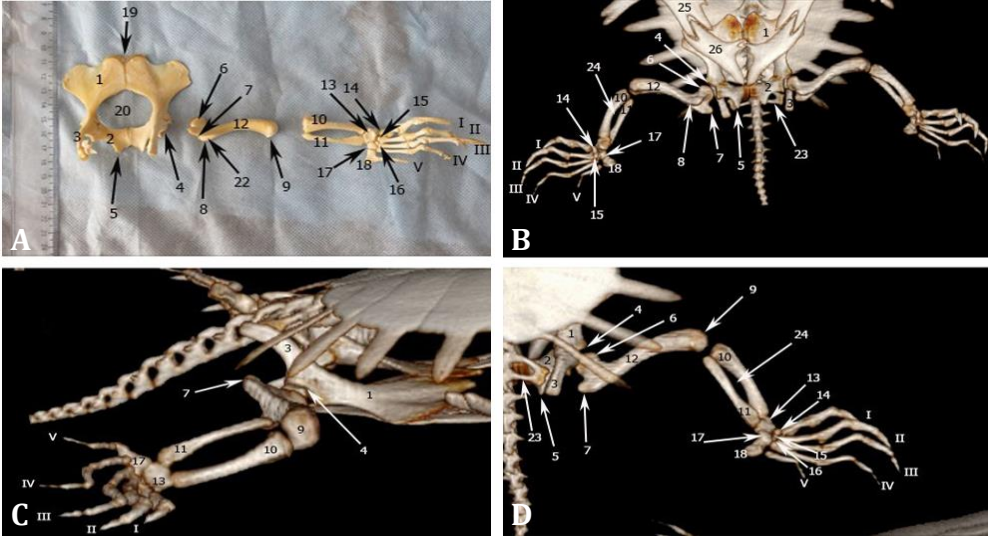
**A)** Dorsal view of bones of the right pelvic limb; **B)** Ventral view of the 3D-CT of right pelvic limb; **C)** Lateral view of the 3D-CT of right pelvic limb; **D)** Dorsal view of the 3D-CT of right pelvic limb. **1.** Pubic, **2.** Ischium, **3.** Ilium, **4.** Acetabulum, **5.** Metischial process, **6.** Femoral head, **7.** Greater trochanter, **8.** Lesser trochanter, **9.** Distal end of femur, **10.** Tibia, **11.** Fibula, **12.** Femur, **13.** Astragalus, **14.** 1^st^ distal tarsal (DT), **15.** 2^nd^ DT, **16.** 3^rd^ DT, **17.** 4^th^ DT, **18.** 5^th^ Metatarsal (MT), **19.** Epipubic, **20.** Thyroid fenestra, **21.** Neck, **22.** Trochanteric fossa, **23.** Sacral rib, **24.** Interosseous space, **25.** Fourth segment of plastron (hypoplastron), **26.** Fifth segment of plastron (xiphiplastron). Roman numerals indicate respective digits

**Pelvic girdle. **Two Pubic bones were joined medially at cranial pelvic symphysis. The pubic symphysis was longer than the ischial one. The bone on the cranial part was wide and paper-shaped, but toward the acetabulum become thicker and cylindrical. It had a wing cranially and a body caudally. In the middle of wing cranial border a crescent-shaped notch was seen. On both sides of the notch, medial and lateral tubercles were seen. There was a cartilaginous structure called epipubis between medial tubercles of two pubic bones. Wing lateral border with its body had a great curvature. The caudal border of wing and the medial border of body were involved in the formation of the thyroid fenestra (Fig. 3A). Two ischium bones were joined medially at caudal pelvic symphysis. Cranial borders of two bones made the caudal border of thyroid fenestra which was almost a straight line. Lateral border was curved and caudal borders of two bones were continuously created an ischiatic arch. Metischial process was seen between lateral and caudal ischiatic borders. Ischiatic dorsal surface that was the location of the viscera was concave and its ventral surface was convex (Fig. 3A). Ilium was extended caudodorsally from the hook-like acetabulum to the carapace. Its respective cranial and caudal borders were convex and concave and lateral and medial surfaces were convex and concave. It had an articular surface at the medial side of its caudalmost part which was articulated with two sacral ribs (Fig. 3A).

**Fig. 4:**
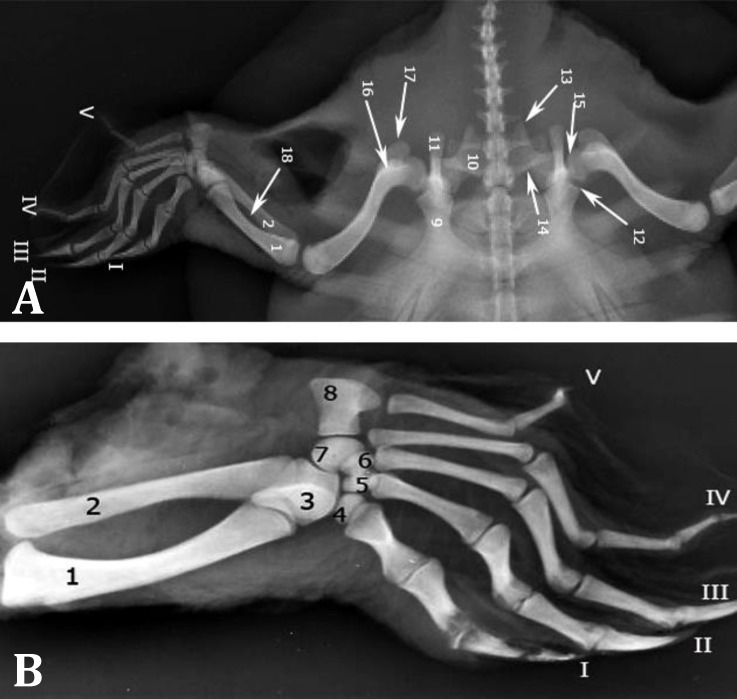
**A)** Dorsoventral radiographic view of the right pelvic limb and **B)** Dorsoplantar radiographic view of left autopodium. **1.** Tibia, **2.** Fibula, **3.** Astragalus, **4.** 1^st^ Distal tarsal (DT), **5.** 2^nd^ DT, **6.** 3^rd^ DT, **7.** 4^th^ DT, **8.** 5^th^ Metatarsal (MT), **9.** Pubic, **10.** Ischium, **11.** Ilium, **12. **Acetabulum, **13.** Metischial process, **14.** Sacral rib, **15.** Femoral head, **16.** Lesser trochanter, **17.** Greater trochanter, **18.** Interosseous space. Roman numerals indicate respective digits

In DV radiographic view, ischium borders were clearly observed. Metischial process that is the most caudal part of the pelvic floor was clear. Cranial borders of ischium made the caudal border of thyroid fenestra. Cranial parts of pubic bones superimposed with 5^th^ segment of plastron (xiphiplastron), but its boundaries were recognized. Acetabulum was also identified at the caudal and lateral side of the radiographs. Ilium in some extent super-imposed by pelvic floor and was extended caudally from the acetabulum (Fig. 4A). In lateral view, ilium and pubis transverse sections were clearly observed.

Due to carapace superimposition, in dorsal view of the 3D-CT images, only ilium, some parts of ischium and the acetabulum were observed (Fig. 3D). In lateral view, some parts of pubis, ilium and the acetabulum were seen (Fig. 3C). In ventral view, some parts of pubis were covered by zyphoplastrone, but ilium, sacral ribs and acetabulum were observed obviously. Ischiatic bones were fused medially to make the ischiatic arch (Fig. 3B).

**Stylopodium. **Femur was a long bone with a mild curvature in the body. Its proximal extremity had an oval articular head for the hip joint. Greater and lesser trochanters were observed on dorsal and ventral sides of the head. A trochanteric fossa was seen caudally between two trochanters. The head was separated from the body by a neck. The body was convex cranially and concave caudally. Distal half of body was flattened and had dorsal and ventral borders. The femur had two articular surfaces in its distal extremity in order to articulate with the fibula and tibia (Fig. 3A). 

In DV radiographic view, femur with its slight curvature was extended laterally. Its head was clearly seen in the acetabulum. Greater and lesser trochanters were seen caudally. Cranial and caudal borders of the shaft were convex and concave, respectively. Distal articular surface was articulated laterally with the zeugopadial bones (Fig. 4A). 

In dorsal view of the 3D-CT images, femur was seen at the caudal border of the carapace and was covered in some extent by it. Greater trochanter was observed. Cranial contour of the shaft was convex and caudal one was concave (Fig. 3D). Lesser trochanter was seen only in ventral view (Fig. 3B). In lateral view, distal and proximal extremities and greater trochanter were identified (Fig. 3C). 

**Zeugopodium.** Tibia and fibula were joined to each other only in their proximal extremities. In distal extremity, each of them was articulated to a separate articular surface of the astragalus. Tibial shaft had three caudal, dorsal and ventral surfaces and cranial, dorsal and ventral borders. The dorsal and cranial borders had a significant curvature and a crest, respectively. The body of tibia and extremities were wider than the fibula. Its proximal extremity was larger than distal one and had a wide articular surface for the femur. Its distal trochlear articular surface was articulated with astragalus (Fig. 3A). Fibula had more delicate body than tibia. In proximal extremity, it had ventral and dorsal articular surfaces and medial and lateral borders. Its lateral border was straight but the medial one was curved. This curvature was seen clearly at the distal extremity. The body close to the distal extremity was triangular and larger than the proximal (Fig. 3A).

In DV radiographic view, tibia and fibula were extended from craniomedial to caudolateral. Slender fibula was seen caudal to the tibia (Fig. 4A). 

In all views of the 3D-CT images, tibia and fibula were seen. Tibia had a wider and shorter shaft compare to fibula and was located cranioventrally. The interosseous space was well-distinguished in lateral view. In the ventral view, some parts of fibula were covered by tibia and were not completely seen (Figs. 3B, 3C and 3D).

**Autopodium.** In this region, tarsal and metatarsal bones and phalanges were seen. Tarsal bones were located in two proximal and distal rows. The bones of proximal row were fused together and made astragalus. There were four bones in the distal row. Astragalus was articulated proximally to fibula and tibia and had fossae on both dorsal and plantar surfaces. The bone was articulated to 1^st^, 2^nd^ and 3^rd^ distal tarsal bones distally and to the 4^th^ distal tarsal bone laterally. Each of distal tarsal bones was located proximal to its corresponding metatarsal bone. Fourth distal tarsal was larger than others and joined to 5^th^ metatarsal bone in addition to 4^th^ metatarsal bone. Five metatarsal bones were seen distal to the tarsus. First to 4^th ^metatarsal bones were long bones that had a body and two extremities. Extremities of the bones were wider than the body. Metatarsal’s width was decreased from 1^st^ to 4^th^. First and 2^nd ^metatarsals were longer and slender than 1^st^ and 2^nd^ metacarpals. Fifth metatarsal had a rectangular shape with a protruded distal articular surface for the first phalanx of the fifth digit. The bone to the manus, the distal phalanges of 1^st^, 2^nd^ and 3^rd^ digits were cone-shaped, ending in a pointed tip. The number of the phalanges of 1^st ^to 5^th^ digits was 2, 3, 3, 4 and 3, respectively (Fig. 3A).

Like the manus in trunk DV radiographic view, auto-podial bones were superimposed and not recognizable from each other. In order to observe these components, positioning was changed and dorsoplantar radiography was done. In this view, all tarsal bones were observed. Astragalus was seen in proximal row and superimposed in some parts with tibia and fibula distal extremities. Its articular surfaces with all 4 distal tarsal bones were clear. Each of distal tarsal bones was seen proximally on its corresponding metatarsal bone. Fourth distal tarsal bone was the largest and articulated to 5^th^ metatarsal bone laterally. First, 2^nd^ and 3^rd^ distal tarsal bones were observed distal to astragalus and 4^th^ distal tarsal bone was on its lateral side (Fig. 4B). Metatarsal thickness and density were decreased from 1^st^ to 4^th^ and 4^th^ metatarsal bone was the longest. Fifth metatarsal bone was quite different from the rest of metatarsals and was seen in the same level as distal tarsal bones. The cone-shaped and sharp ends of the last phalanges of the 1^st^ to 3^rd^ digits were well-cleared (Fig. 4B). Fourth and 5^th^ digits had small distal phalanx. Conical processes of the distal phalanges of the 1^st^, 2^nd^, and 3^rd^ digits were observed (Fig. 4B).

In dorsal and ventral views of the 3D-CT images, all autopodial bones were observed. In lateral view, astragalus and distal tarsal IV were identified. Metatarsals 1, 2, 3 and their corresponding distal tarsal bones were superimposed (Figs. 3B, 3C and 3D). 

## Discussion

Few studies have been conducted on normal anatomical and radiological views of turtles' bones. Sheil studied bones' morphogenesis during embryonic period in soft shell adult turtle (*Apolone spinifera*).^6^ Valente *et al*. described radio-graphic anatomy of the limbs of the loggerhead sea turtles (*Caretta* *caretta*).^3^ The researchers used 3D-CT images in their description. In other studies which evolutionary anatomic aspects of the bones have been presented, usually direct study of bones has been used.^8^^-^11 In this study, anatomy, radiology and 3D-CT of limbs' skeleton were studied in the Euphrates turtle species for the first time. Radiography and 3D-CT helped us in identifying correct direction, angle and position of the bones. Regarding small bones that inevitably were disjoined from each other in bones' preparation, radiography and 3D-CT scans were very helpful in assembling the skeleton.

Knowledge of normal radiography of bones is essential for proper diagnosis of bones' related problems and diseases. In most cases, clinical examination does not provide sufficient information about referred turtles to the clinic and radiography is one of the best paraclinical diagnostic methods. Radiography provides an overview of the skeleton and is a noninvasive and inexpensive method to locate turtles' fractures.^2^ On the other hand, CT solves problems due to superimposition and is used to detect fractures, dislocations, osteoporosis and neoplasia.^4^ In the present study, simultaneous anatomical, radiographic and CT studies of bones on individual turtles made it possible to describe bones anatomically and provided comparable and complementary conditions to represent the abilities of the radiography and CT for a better understanding of the anatomy. For instance, anatomical study of the distal extremities of radius and ulna and their positions to carpal bones clearly showed why in dorsopalmar radiography of the autopodium, superimposition of these bones occurred.

Arrangement and the number of carpal and tarsal bones, in turtles' family have been significantly changed during evolution.^8^ These changes have occurred to the extent that autopodial anatomy is used in turtle’s classification. It has been said that carpal morphology diversity is more than tarsal one. The most reported autopodial variations in turtles include fusion of 3^rd^ to 5^th^ distal carpal, or only 4^th^ and 5^th^; the number of central bones; presence or absence of pisiform bone; and fusion or separation of astragalus and calcaneous.^9^ In anatomical studies that have been conducted on limbs of different species of turtles, autopodial region has been noted mainly.^6^^,^^8^^,^^10^^-^^13^ The shape and location of the 5^th^ metatarsal bone in Euphrates turtle can be due to embryonic fusion of 5^th^ distal tarsal to the 5^th^ metatarsal bone. However, among the studied species, Euphrates turtle carpal and tarsal bones show the most similarities to the turtle* Apolone spinifera*. Table 1 shows the number of autopodial bones of the Euphrates and other studied turtle species.

**Table 1:** Autopodial formula of some turtle species

**species**	**Proximal** **row** **of carp**	**Distal row** **of carp**	**Manus phalangeal formula** *	**Proximal** **row** **of tars**	**Distal row of tars**	**Pedis phalangeal formula** *	**Reference**
***Apolone spinifera***	5	5	2.3.3.4.3	1	4	2.3.3.4.2	6
***Podonemis expansa***	5	5	2.3.3.3.3	2	5	2.3.3.3.3	8
***Macrochelys temmincki***	5	5	2.3.3.3.3	1	5	2.3.3.3.2	10
***Clydra serpentina***	5	5	2.3.3.3.3	1	5	2.3.3.3.2	11
***Phrynops hillari***	5	5	2.3.3.3.3	1	4	2.3.3.3.5	12
***Pelodiscus sinensis***	5	5	2.3.3.5.4	1	4	2.3.3.4.3	13
***Emydura subglobosa***	5	5	2.3.3.3.3	2	5	2.3.3.3.2	14
***Rafetus euphraticus***	5	5	2.3.3.5.4	1	4	2.3.3.4.3	Present study

* Medial to lateral.
